# Why there are so many definitions of fitness in models

**DOI:** 10.1093/genetics/iyag090

**Published:** 2026-04-27

**Authors:** Daniel J B Smith, Guilhem Doulcier, Pierrick Bourrat, Peter Takacs, Joanna Masel

**Affiliations:** Ecology & Evolutionary Biology, University of Arizona, Tucson AZ 85721, United States; Department of Integrative Biology and the Oden Institute for Computational Engineering and Sciences, The University of Texas at Austin, Austin, TX 78712, United States; School of Humanities, Philosophy Discipline, Macquarie University, Macquarie Park, NSW 2109, Australia; Theory Department, Max Planck Institute for Evolutionary Biology, Plön 24306, Schleswig-Holstein, Germany; School of Humanities, Philosophy Discipline, Macquarie University, Macquarie Park, NSW 2109, Australia; Charles Perkins Centre, University of Sydney, Sydney, NSW 2006, Australia; School of Humanities, Philosophy Discipline, Macquarie University, Macquarie Park, NSW 2109, Australia; Ecology & Evolutionary Biology, University of Arizona, Tucson AZ 85721, United States

**Keywords:** invasion fitness, Malthusian parameter, individuality, theoretical population genetics, bet hedging, life history strategy, density-dependent selection

## Abstract

Evolutionary “fitness” is operationalized in many different ways in models. Its role is to quantify that which is favored by natural selection. Generally, short-term ability to survive and reproduce (e.g. expected number of surviving offspring) is *assigned* to genotypes or phenotypes and used to non-trivially *derive* longer-term quantities (e.g. invasion rate or fixation probability) that provide insight as to which organismal strategies tend to evolve due to natural selection. Assigned fitness operationalizations either explicitly or implicitly specify organismal vital rates (i.e. births, deaths, organismal growth). Derived operationalizations also depend on assumptions regarding demographic stochasticity; environmental stochasticity; feedbacks whereby births, deaths, and organismal growth cause environmental change; and the impact of migration and niche construction on which environment is experienced. The choice of derived operationalization can impact conclusions, as we illustrate for the evolution of bet hedging when treated by invasion probability vs expected Malthusian parameter within an adaptive dynamics approach. After reviewing existing derived fitness operationalizations, we propose a new one that meets the particular challenges posed by balancing selection. Population genetic models generally sidestep ultra-high-dimensional phenotype and genotype spaces by deriving the long-term evolutionary fate/fitness of a lower-dimensional set of genetically encoded “strategies.” Strategies (e.g. costly developmental commitment to producing armaments) are causally upstream from realized phenotypes (e.g. armament size), but downstream from how an organism's early environment (e.g. maternal effects) might inform developmental commitments. While selection is best understood in terms of differences in *organismal* vital rates, its derived outcomes are most easily understood as properties of *genetic lineages*.

## Teaser

This manuscript argues that what unites the surprisingly diverse range of mathematizations of evolutionary “fitness” is an intent to quantify what is favored by natural selection. Each evolutionary theory paper assigns one or more fitness-like quantity to genotypes or phenotypes, from which it non-trivially derives one or more longer-term quantities. Smith et al. argue that organismal vital rates are the ultimate assigned fitness-like quantities, and the-long term fate of biological strategies is best captured by the derived fate of genetic lineages. However, different questions about biological strategies call for different ways of defining fitness.

## Introduction

“*Fitness: Something everyone understands but that no one can define precisely.*” (Stearns 1976)

Darwin's theory of evolution by natural selection did not launch a professional discipline of evolutionary biology until the modern synthesis of the early twentieth century, in which the role of mathematical population genetics was key (Provine 1978). Central to this mathematization was “fitness,” which turned Darwin's intuitions about “what tends to be favored in the struggle for existence” into more formal quantitative operationalizations of the ability to survive and reproduce. Resulting models are used to derive non-obvious insights (Servedio et al. 2014). Here, we focus primarily on the use of models to deduce what natural selection is likely to favor. Fitness models are also used to deduce results about the timescale of evolution (Provine 1978; Charlesworth 2020); we cover this to a lesser degree. Fitting models of fitness to sequence data (e.g. to detect loci under recent selection (Enard 2021)) falls outside the scope of the current, more theoretically focused manuscript.

From the outset of its mathematization, fitness has been operationalized in different ways (Ariew and Lewontin 2004; Orr 2009). We use the term “fitness operationalization” to refer very broadly to any quantity that a model uses to define and/or summarize survival, growth, reproduction, and/or persistence among types, at any timescale. Haldane (1927) used the expected *absolute* number of surviving offspring, while the influential Wright–Fisher model used the expected *relative* contribution to the gene pool in the next generation (Fisher 1930; Wright 1931). Theoretical population genetic models *assign* some version of expected short-term fitness to genotype–environment combinations, from which they mathematically *derive* longer-term outcomes. For example, Haldane (1927) assigned births per generation to a mutant genotype and then derived its probability of fixation. Each such model thus involves at least 2 fitness operationalizations: the assigned short-term fitness and the derived long-term outcome or probability distribution of outcomes.

We use annual plants and the Hawk–Dove game (Maynard Smith and Price 1973) as illustrative examples to review a variety of models and corresponding fitness operationalizations, their motivation, and simplifying assumptions. We first focus on operationalizations that are at least sometimes assigned, then on operationalizations that are always derived. We then propose a conceptual scheme describing how models give insights into the fates, under natural selection, of the organismal strategies being investigated. We argue that short-term fitness is best operationalized via *organismal* vital rates (births, deaths, organismal growth) plus organismal effects on the experienced environment through migration (i.e. selection of local environment by organism) and/or niche construction (i.e. modification of local environment). In contrast, what natural selection favors in the long term is best operationalized via *genetic lineages*.

## Assigned fitness

### Absolute fitness

Absolute fitness *W* describes the expected number of surviving offspring that a (hermaphroditic or asexual) individual produces after reaching reproductive maturity. Equivalently, it describes a juvenile's expected number of offspring (reversing the order of survival and reproduction). Either way, it is the expectation over one complete life cycle or “generation” of both survival and reproduction.

**Fig. 1.**
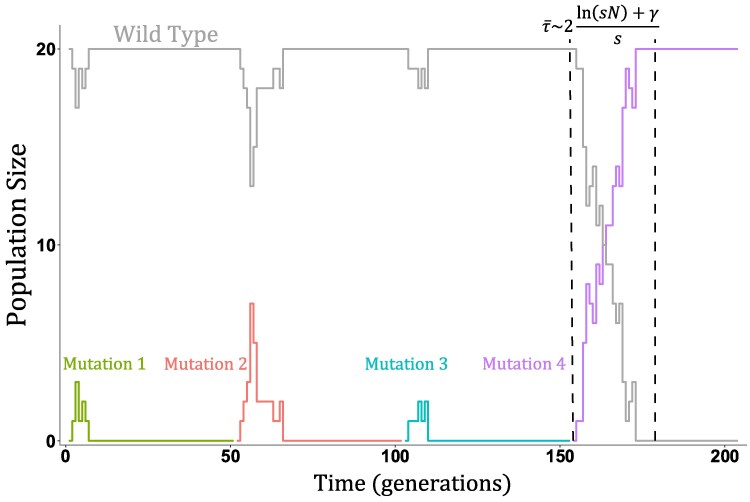
Fixation probability and sojourn time capture different long-term consequences of natural selection. Representative Wright–Fisher simulation of a population of size N=20 in which an allele with selection coefficient s=0.125 appears repeatedly by mutation. The mutant fixes with probability $\approx 2s/\sigma^{2}=0.25$. The sojourn time *τ* describes the number of generations before a mutation fixes (given it does not go extinct) with mean $\bar{\tau }=2(\ln\;(sN)+\gamma )/s$. Each color indicates a different mutation. The interval between the dashed lines depicts $\bar{\tau }$, which is slightly shorter than the realized value of *τ* in this simulation.

The seminal use of absolute, per-generation fitness was to assign absolute fitness and to derive from it the probability of fixation (or, using more recent and precise terms, the probability of “invasion” or “establishment”) of a new beneficial mutation. Haldane (1927) considered a resident (*R*) population of constant size, such that $W_{R}=1$. He then considered the fate of a new lineage produced by a beneficial mutation. Individuals carrying the mutation have $W_{I}=1+s$, where the selective advantage s>0. With some simplifying assumptions, including a Poisson distribution of offspring and s≪1/2, Haldane (1927) derived the probability that the beneficial mutation escapes extinction to “invade” as 2s (Fig. 1). Beyond the Poisson distribution, an exact branching process approach can be used to show that the invasion probability for small *s* is $2s/\sigma^{2}$, where $\sigma^{2}$ is the per individual variance in offspring number (Barton et al. 2007, p. 25; Leigh 1990, p. 176).

This example illustrates how the long-term fate of a mutant (probability of invasion) is derived from short-term probability distributions of offspring number. Evolutionary success under natural selection cannot be reduced, even in a very simple model, to a single number such as *W* (Krimbas 2004). Larger variance in reproduction $\sigma^{2}$ increases the extinction probability, which can loosely be understood in terms of a lower signal (s) to noise $(\sigma^{2}$) ratio.

Haldane's assignation of the expected absolute number of surviving offspring (fitness *W*) to genotypes is rarely used outside of this example of a rare beneficial mutant. All biological populations are density regulated, meaning that high *W* causes an increase in population density, which in turn reduces *W* (Haldane 1956; Nicholson 1957). For assigned constant *W*, the invading mutant lineage instead experiences unbounded exponential growth.

### Relative fitness

Assigning *relative fitness* instead of *absolute fitness* sidesteps the issue of unbounded exponential growth. Relative fitness models treat the proportions of variants, rather than their absolute abundances. To motivate this, Crow and Kimura (1970, pp. 25–26) derived relative fitnesses $w_{i}=\frac{W_{i}}{\bar{W}}$ from assignations of absolute fitnesses $W_{i}$ in the context of exponential population growth or decline. On this basis, they argued for simplified models in which $w_{i}$ rather than $W_{i}$ values are directly assigned. Measurement theory has also been invoked to support the use of relative fitness over alternatives (Wagner 2010).

In these simplified models, which have become standard within population genetics, relative fitness is defined as proportional to the expected fraction of the next generation that is descended from the focal genotype or individual. In practice, relative fitnesses are assigned as unique quantitative values relative to a “wild-type” fitness of 1. In the simple case of asexual reproduction, if $p_{i}(t)$ is the proportion of the population with genotype *i* at time *t* and $w_{i}$ is the assigned relative fitness of type *i*, then its expected proportion in the next generation is


(1) $$\;\;p_{i}(t+1)=p_{i}(t)\frac{w_{i}}{\bar{w}}.\;$$

To keep the population size constant, normalization by the population mean of the arbitrarily scaled relative fitness $\bar{w}=\sum_{\mathrm{all}\;gti}\;p_{i}(t)w_{i}$ derives absolute fitness $W_{i}=\frac{w_{i}}{\bar{w}}$ from assignations of fitness relative to the wild type. Alternative demographic models, e.g. exponential growth, produce different absolute fitness.

A classic use of relative fitness assignations is in Wright–Fisher models that select among parent genotypes whose expected fecundity is $w_{i}/\bar{w}$. All adults then die—a potentially appropriate model for an annual plant. The finite population size *N* of a Wright–Fisher model enables the derivation of fixation probabilities also for deleterious mutations, which never avoid extinction under the branching process treatment of Haldane (1927). Finite population size also enables derivation of the expected “sojourn” time $\bar{\tau }$ prior to extinction or fixation (Ewens 1964; Kimura and Ohta 1969; Charlesworth 2020). Conditional on fixation, $\bar{\tau }∼2(\ln\;(sN)+\gamma )/s$ generations in a haploid Wright–Fisher model (Fig. 1) where γ≈0.5772 is Euler's constant (Hermisson and Pennings 2005). Sojourn times are one way to clearly show that natural selection works sufficiently rapidly to be a major cause of evolution, a historically important influence of mathematical theories on biological thought (Provine 1978; Charlesworth 2020). In the modern era, the duration of sojourn at each allele frequency is used when inferring a population's history of selection and demography from sequence data (Gutenkunst et al. 2009; Keightley and Halligan 2011; Ronen et al. 2013; Charlesworth 2020; Liu and Fu 2020; Excoffier et al. 2021).

A key limitation of models that assign relative fitness to genotypes is that they do not allow the consequences of selection to feed back onto population density. In other words, the population size *N* is externally set, independently of mean population fitness. Problematically, no matter how low population fitness *w* drops, the externally set population size *N* will not decline, contradicting the biological intuition that low fitness should indicate an increased tendency to go extinct. A second, related limitation is that relative fitness cannot be compared across populations.

### Vital rates are the “ultimate” assigned values

Vital rates describe rates of organismal growth, deaths, and reproduction. To complete a generation, seeds must germinate and survive to become seedlings, then survive from seedlings until they reach reproductive maturity, and then produce and disperse seeds. This description of 3 “fitness components” encompasses 3 vital rates for 3 life history transitions: the first 2 include both survival and growth, while the third includes only reproduction. Per-generation absolute fitness is the product of fitness components, each describing survival and/or reproduction during a different life history transition, within a fixed sequence. However, when the sequence varies, different values of fitness components are derived from the same vital rates, e.g. for a seed that survives within a seed bank for a variable number of years, each time without growth.

Like Metcalf and Pavard (2007), Doebeli et al. (2017), and Matheson et al. (2025), we propose making survival and reproduction core to our scheme by assigning corresponding rates of death *d* and birth *b* (either in continuous time or discrete per-generation), rather than values of “fitness,” to phenotypes in an environment. On the surface, many models assign relative or absolute fitness values. However, a classic model such as Wright–Fisher is better seen as assigning a variable rate *b* of births/generation combined with a constant adult time to death of 1/d=1 generation. From these vital rate assignations, per-generation fitness is implicitly and trivially derived as W=b/d (*b* births per lifespan of length $\frac{1}{d}=1$). *W* in Haldane (1927) can similarly be seen as a derived fitness operationalization from implicit assignations of *b* and *d*, where a variety of assignation scenarios can generate the same values of *W*, including scenarios of stochastic births and deaths in continuous time. In more complex models, e.g. of populations perturbed away from demographic equilibrium, selection on fecundity and/or juveniles does not produce the same allele frequency trajectory as selection on adult death rates (Benton and Grant 2000; Bertram and Masel 2019a).

When describing vital rates as *organismal*, we remain agnostic regarding the thorny question of individuality (Clarke 2010; Lidgard and Nyhart 2017; Bourrat 2023; Wilson and Barker 2024). For example, a yeast cell division event can be seen as a birth event from the perspective of individual cells, but a growth event from the perspective of individual colonies. From either perspective, it is an organismal vital rate, and our scheme does not require us to adjudicate questions of individuality.

It is possible to extend the organismal concept of vital rate to the genic level, which might be helpful, e.g. when investigating the evolution of selfish genetic elements such as gene drives or transposable elements. Copies of an allele can be replicated through a variety of means: organismal replication (at different organismal levels of organization), duplication, or gene conversion. Copies of a gene can be lost through either organismal death or deletion/destructive mutation. Longer-term gene-level advantages of selfish elements can be derived from these gene-level vital rates.

## Derived fitness operationalizations

The fitness operationalizations presented so far have a history of being assigned to genotypes (as a function of their current environment). Next, we consider properties that are rarely if ever assigned, but instead derived from assigned fitness operationalizations. To illustrate them, we add a seed bank to our annual plant example.

### The Malthusian parameter

The Malthusian parameter (Malthus 1798; Fisher 1930) or intrinsic growth rate *r* (Lotka 1907) quantifies how quickly a genetic lineage tends to grow or shrink, in absolute time units (e.g. days), rather than in the per-generation time units of the relative and absolute fitness operationalizations above. While usually specified as a form of absolute fitness, a relative fitness version can be obtained as $r_{i}^{\prime }=r_{i}-\bar{r}$, where $\bar{r}\;$ is the mean Malthusian parameter, with $r_{i}^{\prime }$ analogous to $w_{i}/\bar{w}$ discussed above. While *r* occasionally seems to be assigned (Desai and Fisher 2007), such models generally entail additional assumptions about the behavior of birth and death rates, with implications regarding the derivation of *r*.

For the non-overlapping generations treated by the Wright–Fisher model, *r* and *W* contain the same information, albeit in different units. However, consider a simple scenario of overlapping generations, where individuals produce offspring at rate *b* and die at rate *d*. The Malthusian parameter is then trivially derived from the assigned vital rates as r=b−d. Expected per-generation absolute fitness is derived, assuming birth and death are Poisson processes, as W=b/d (expected births occurring during a lifespan whose mean length is 1/d). For example, when b=0.2 and d=0.1, then W=b/d=2 (average of 2 offspring per generation), while r=b−d=0.1 (lineage is growing with exponential growth rate 0.1 per external time unit such that $y(t)=y(0)e^{rt}$). When generations overlap, neither *W* nor *r* can be derived given information only about the other, and they provide information about different things (De Jong 1994; Brommer et al. 2002). The Malthusian parameter tells us what allele frequencies to expect at a specified time in the future (*r* is a *rate*). For example, sojourn time (Fig. 1, $\bar{\tau }$ is shown for mutation 4) depends on differences in *r*, whereas differences in *W*, combined with $\sigma^{2}$ (variance in per-individual lifetime reproductive success), tell us the *probability* that a rare beneficial mutation will escape initial stochastic extinction (Fig. 1, mutations 1 to 4).

Selection can act on differences in one quantity (*r* or *W*) even given equality for the other. For example, consider a trade-off between *b* and *d* such that W=b/d remains constant. Importantly, *r* need not be constant under this constraint. In the wake of a disturbance that kills many individuals from a population previously at equilibrium, selection will favor larger *b* and *d*, because this increases r=b−d, enabling the type with the faster life history to more quickly rise back up to carrying capacity (Stearns 1992). When the mutation rate is fast relative to sojourn time, in what Desai and Fisher (2007) called the “multiple mutations” regime, the faster life history type also has an advantage during clonal interference, even at demographic equilibrium. The degree to which selection and density-regulation act on deaths vs births has implications for *r* and for generation time, but not for *W* (Draghi et al. 2024). Shorter generation time yields higher evolvability, both with respect to selection (Draghi et al. 2024) and to mutation (Verin et al. 2017). Whether *W* vs *r* best predicts invasion depends on which life history stage density is regulated at (Mylius and Diekmann 1995).

Unlike *W*, the Malthusian parameter generally depends on all 3 kinds of vital rate, i.e. on growth as well as on deaths and births. For example, consider adult plants (*A*) that die at rate *d* and give birth at rate *b* to seeds (*S*) that grow into reproductively mature adults at rate *m*. For simplicity, we neglect seed death. This yields the following differential equations:


$$\begin{array}{c} dS(t)/dt\;\\ dA(t)/dt \end{array}=\left(\begin{array}{cc} -m & b\\ m & -d \end{array}\right)\left(\begin{array}{c} S(t)\\ A(t) \end{array}\right)$$

The Malthusian parameter is the dominant eigenvalue of the 2×2 matrix above: $r=\frac{1}{2}\left(\sqrt{4bm+d^{2}+m^{2}-2dm}-d-m\right)$. This equation illustrates the need to include *m*, noting that r→b−d only in the special case as m→∞. In contrast, even in this stage structured model, we still derive *W* equal to b/d with no dependence on *m*.

A common use of the Malthusian parameter is to describe “invasion fitness,” meaning whether and at what speed a new mutant genotype *I* deterministically invades a population of “resident” genotype *R* at equilibrium abundance $\hat{N_{R}}$ (Metz et al. 1992). To illustrate this, consider an annual plant population in which a seed germinates with probability *g* per year to produce an expected *f* seeds, or else survives with probability 1/d in the seed bank. Now our vital rates are *f*, *g*, and *d*. In external timesteps t=1 (rather than per-generation), types i=R,I (resident and invader) obey:


(2) E[Ni(t+1)]=(1/d)(1−gi)Ni(t)⏟Numberofnon-germinatingseedsthatsurvive+Nifgi11+α∑alljgjNj(t)⏟Newseedsproducedbygerminatingindividualsthatsurvivedensityeffects,

where we capture the dependence of fecundity on seedling density using parameter *α*, as is common practice in density-dependent annual plants models (Watkinson 1980; Ellner 1987; Stouffer 2022). For a rare invader ($N_{I}(0)\ll \hat{N_{R}}$), invasion fitness is equal to the absolute Malthusian parameter:


(3) $$r_{I}=E\left(\ln\frac{N_{I}(1)}{N_{I}(0)}\right)\;\;$$

which depends on resident density $\hat{N_{R}}$ via the denominator in the rightmost term of Equation (2). Invader *I* tends to invade if and only if $r_{I}>0$.

So far, the optimal strategy is always to germinate, i.e. $r_{I}>0$ if and only if $g_{I}>g_{R}$. This is because there is so far no advantage to being dormant, to offset the risk of dying while in the seed bank. This changes when we consider fluctuating environments below, in which germination is sometimes futile.

### Fitness across a variable environment

Most organisms experience environmental heterogeneity that affects their vital rates. For example, plant seed production *f* depends on abiotic environmental factors (e.g. rainfall), biotic density-dependent environmental factors (MacArthur 1962; Tilman 1982; Travis et al. 2023), and biotic frequency-dependent environmental factors (Tilman et al. 2020). The social environment (e.g. pollinators and/or interference competition) is included within the biotic density-dependent and frequency-dependent factors. Environmental variation can be spatial and/or temporal.

Given spatial environmental variation, migration enables organisms to affect which environment(s) they encounter. Some forms of migration, e.g. seed dispersal, are closely coupled to a life history transition, but can be conceptually separated into a migration phenotype in the old location, followed by vital rates of birth, death, and growth in the new location. Similarly, organisms can indirectly modify their vital rates via phenotypes that physically alter their local environment (niche construction; Odling-Smee et al. 1996). Selection on migration and niche construction phenotypes is included within the Malthusian parameter calculated across spatial environmental variation. That is, the Malthusian parameter is derived not just from assigned vital rates, but also from assigned migration and niche construction rates.

We consider temporal variation in the environment e(t) via an extension of Equation (2) in which germinating seeds produce zero offspring during drought years, such that fecundity


$$f(e(t))=\{\begin{array}{c} \;f\;\mathrm{in}\;\mathrm{good}\;\;\mathrm{years}\;\;\mathrm{with}\;\mathrm{probability}\;p\\ 0\;\;\mathrm{in}\;\mathrm{bad}\;\mathrm{years}\;\;\mathrm{with}\;\mathrm{probability}\;1-p \end{array}.$$

Instead of the instantaneous Malthusian parameter in a single environment, we take, as invasion fitness, its expected value across the distribution of environments e(t):


(4) $$\;r_{I}=E_{e(t)}\left(\ln\frac{N_{I}(t+1)}{N_{I}(t)}\right).$$

This is known as the *geometric mean fitness* because it corresponds to the geometric mean of absolute per-generation or per-time-step *W* (Yoshimura and Jansen 1996). It is equivalent to the arithmetic mean of the Malthusian parameter over environments (Takacs and Bourrat 2022, 2024). Using the geometric mean of relative fitness can give problematic results; the appropriate geometric mean is that of absolute fitness (Kim 2023), e.g. following normalization in Equation (1). In more complex scenarios when multiple life stages are affected by the environment, a generalization of the Malthusian parameter known as the Lyapunov exponent can be used (Cohen 1979; Metz et al. 1992; Kussell and Leibler 2005).

While germination probability g=1 maximizes $r_{I}$ in a constant environment, it results in complete extinction in a bad year, and so a more conservative $g_{I}<1$ maximizes $r_{I}$ in a temporally varying environment. This is an example of evolutionary *bet hedging* (Cohen 1966; Seger and Brockmann 1987; Frank 2011a).

In adaptive dynamics (Metz et al. 1995), the standard practice is to assume that evolution moves in the direction that maximizes invasion fitness given infinitesimal perturbations to parameters controlling strategies (e.g. $g_{I}$ infinitesimally differs from $g_{R}$). In the seed bank model, evolved $g_{R}$ then achieves $r_{I}<0$ for all $g_{I}\neq g_{R}$ (an “evolutionary stable strategy”; Geritz et al. 1998). However, the probability that an invader escapes initial stochasticity cannot be predicted from invasion fitness $r_{I}$ alone (Yoshimura and Jansen 1996), contradicting standard assumptions within the adaptive dynamics field.

### Fixation probability ratio

Derived fitness operationalizations attempt to capture which strategies will become prevalent, if present, as a consequence of natural selection. Although individuals die within a short timescale, they embody a strategy/type (e.g. tendency to dormancy) subject to genetic inheritance such that it can last over a longer timescale. Many models use a “phenotypic gambit” to simplify the relationship between genotype and strategy by assuming that strategies are encoded by variant alleles at a single locus. Other models make the simplifying assumption that only one variant is introduced at a time, also allowing recombination between variants to be neglected. The evolution of these strategies/types is then exactly captured by lineage-based fitness operationalizations that track the fate of mutations at 1 locus.

The concept of genetic lineages (Akçay and Van Cleve 2016; Graves and Weinreich 2017) can be expanded beyond these simplified scenarios. We define a genetic lineage as all gene copies descended from a new mutation, e.g. encoding a change in germination probability. Separate lineages can be founded by independent mutations of the same allele. Sublineages that are still part of the original lineage can be created by a subsequent reversion mutation to the ancestral allele or a new mutation to a third allele. Due to recombination, different genetic lineages at different loci are nested within a common organismal genealogy (Kelleher et al. 2018). A lineage can even cross species boundaries following a horizontal gene transfer event. In the long term, each lineage either fails (goes extinct) or succeeds (fixes in the population). Under this expanded concept of genetic lineage, the probabilities of lineage fate can be used to construct a derived operationalization of fitness.

In contrast to the probability of lineage fate, invasion fitness (equations 3 to 4), by equating $r_{I}>0$ with success, neglects chance extinction. Recalling that the probability of invasion is $∼2s/\sigma^{2}$, invasion fitness does nothing to capture genetic variation affecting demographic stochasticity $\sigma^{2}$. Stochasticity in the series of environments also contributes to extinction (King and Masel 2007; Libby and Ratcliff 2019).

Consider an extension of the annual plant example in which genotype abundance is a discrete random variable *X*:


(5) $$\;\;\;\;\;\;\;\;\;\;N_{i}(t+1)=X(\mu ,\sigma^{2}).$$

Equation (2) on its own is sufficient to describe only the special case with $\sigma^{2}=0$. As in Haldane (1927), $N_{i}(t+1)$ can be 0 even if $\mu >N_{i}(t)$.

**Fig. 2.**
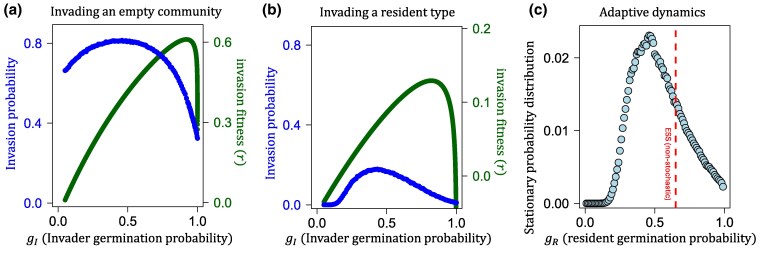
Selection for invasion probability yields a lower germination probability than does selection for invader geometric mean growth *r*. Each year allows reproductive success with p=0.95, and 1/d=.95,f=2.25,α=0.075 throughout. An invader with germination probability $g_{I}$ is introduced into an empty community a) or a resident population of $g_{R}=0.2$ b). Note the different *y* axis scales in green, with negative *r* possible relative to a resident but not relative to an empty community. Invasion probability (blue) is defined in a) as the probability that an invader persists for at least 20 generations and in b) as the probability that the resident goes extinct before the invader does. Invasion fitness *r* (green) peaks at $g_{I}∼0.8$, but invasion probability (blue) is highest for moderately low values of $g_{I}$. In b), $g_{I}<g_{R}=0.2$ yields negative $r_{I}$ and invasion probability ≈0. Note that $r_{I}$ peaks at smaller $g_{I}$ in b) than in a)—this reflects how density dependence affects optimal germination rate (Bulmer 1984; Gremer and Venable 2014; Kortessis and Chesson 2019). c) Long-term evolutionary outcomes. Akin to adaptive dynamics models, we simulate a single resident type with germination probability $g_{R}$ competing against 2 invading lineages with germination probabilities $g_{R}\pm 0.01$. With traditional adaptive dynamics, the lineage with higher $r_{I}$ is chosen deterministically, based on a probability distribution for the series of environments. The evolved germination frequency under demographic stochasticity (shown as a stationary distribution of $g_{R}$) is lower than the evolutionary stable state (ESS) of $g_{R}$ given by adaptive dynamics (dashed red vertical line). We calculate the stationary distribution from a tridiagonal matrix specifying probabilities of transitioning between 2 adjacent germination probabilities $0.005\leq g_{R}\leq .995$, treated in increments of 0.01. We simulated pairs of transition probabilities under both demographic and environmental stochasticity by simultaneously introducing 1 individual of each of 2 invader types via mutation with germination probabilities $g_{R}+0.01$ and $g_{R}-0.01$. To set the initial number of resident individuals, we ran a simulation of the resident type alone for 100 generations and used its final abundance. To avoid chance extinctions of the resident during these burn-ins, we used a reflecting boundary. We perform $5\times 10^{4}$ simulations for each $g_{R}$, then derive the stationary probability distribution of $g_{R}$ as the leading eigenvector of the transition matrix. The density dependence term *α* partially determines the emergent population size *N*. Adult population size varies with $g_{R}$ between simulations, where $\bar{N}∼80$ and $\bar{N}∼25$ for low and high $g_{R}$, respectively. We chose values of *N* this low to exaggerate demographic stochasticity for the purpose of illustration.

The distinction between invasion fitness and invasion probability has substantive consequences, e.g. when predicting which strategies are favored by selection in a finite population experiencing environmental variation in adult mortality (Proulx and Day 2002). In the case of bet hedging, the probability of invader lineage fixation is maximized at a lower value of $g_{I}$ than the maximal geometric mean growth rate is (Fig. 2a and b). Larger *g* causes greater fluctuations in *N*, which means that increasing *g* to maximize invasion fitness *r* has the side effect of reducing the persistence time of a population and/or the sojourn time before loss of somewhat stable coexistence (Adler and Drake 2008; Gourbière and Menu 2009; Okabe and Yoshimura 2022). Beyond pairwise fitness comparisons, demographic stochasticity can also modify mean evolved trait values (Lande 2007; Gourbière and Menu 2009; DeLong and Cressler 2023). In our seed bank example, iteratively choosing invaders based on fixation probability rather than on *r* produces a lower evolved value of *g* (Fig. 2c). Invasion fitness *r* thus does not fully capture the long-term fates of genetic lineages (Constable et al. 2016), including those representing introduced species (Pande et al. 2020, 2022).

Fixation of a beneficial variant can be partitioned into “establishment” (reaching high enough abundance such that deterministic dynamics dominate) vs subsequent competitive superiority over competing established lineages (Desai and Fisher 2007). The relative importance of establishment probability vs invasion speed *r* in determining the outcome of adaptive evolution (i.e. successful fixation) depends on which parameter value regime a population is in. When adaptive mutations are rare, the rate of adaptive substitutions depends on the fixation probability times the beneficial mutation rate, but not at all on the invasion speed (Yampolsky and Stoltzfus 2001). The same is true when recombination is common relative to adaptive mutations, such that each sweep occurs independently, with no clonal interference. When adaptive mutations are common, creating strong clonal interference, the invasion speed becomes more important, albeit not exclusively so (Gomez et al. 2020).

We can assess fixation probabilities as a kind of derived fitness operationalization by comparing them to those of neutral alleles (Nowak et al. 2004). To more fully capture their impact on long term evolutionary outcomes, we can use the ratio of the probability with which allele 1 invades a population in which allele 2 is resident: the probability with which allele 2 invades a population in which allele 1 is resident (Masel 2005). When mutation between the 2 alleles is symmetric and rare, the fixation: counterfixation ratio describes the odds with which a population will be found fixed for allele 1 vs allele 2. This makes it directly applicable to empirical situations such as quantifying preferences among codons (Bulmer 1991; Weibel et al. 2024), in which there is sufficient data across an ensemble of comparable instances.

Note that when mutation is not symmetric, the direction and degree of mutational asymmetry also affect the odds with which a population will be found fixed for allele 1 vs allele 2, which are given by $\mu_{\;j\to i}p_{\mathrm{fix}}(\;j\to i)\;:\mu_{i\to j}p_{\mathrm{fix}}(i\to j)$. This ratio includes both our fitness operationalization $p_{\mathrm{fix}}(\;j\to i)\;:p_{\mathrm{fix}}(i\to j),\;$ and a mutation bias term $\mu_{\;j\to i}\;:\mu_{i\to j}$. The relative mutation rates matter because a variant must first appear in the population before it can be subject to natural selection. We here include as fitness operationalizations anything that quantifies “what evolution tends to favor,” but do not include still broader quantifications of “what evolution tends to make prevalent,” because natural selection is not the only cause of directional evolution (Stoltzfus and Yampolsky 2009).

The evolved mutation rate is a good example of an outcome determined in part by mutation bias. There are more mutations that increase the mutation rate (mutators) than decrease it (antimutators). However, indirect selection against deleterious mutation load favors a lower mutation rate (Johnson 1999a, 1999b), which can result in a mutation-selection-drift balance (Lynch 2008). Using the ratio of fixation: counterfixation probabilities as a fitness operationalization readily handles the complexities of indirect selection that arise e.g. during the evolution of mutation rate.

### How do we operationalize fitness under balancing selection?

**Fig. 3.**
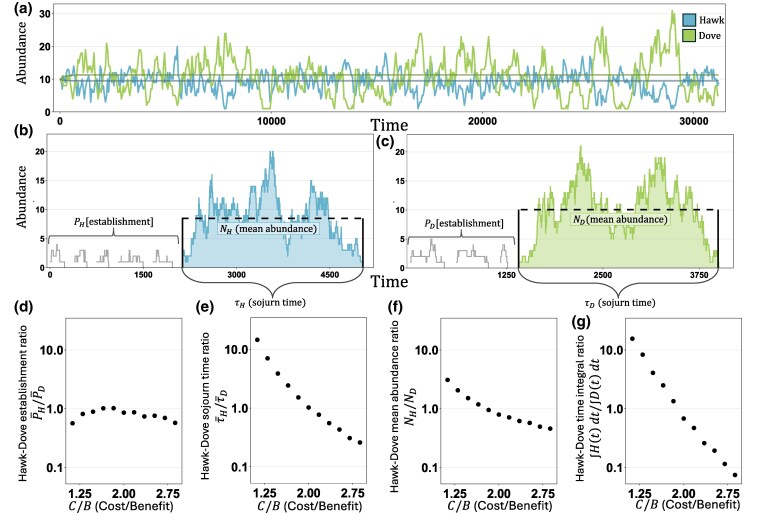
Our proposed operationalization of long-term fitness for a balanced polymorphism. We simulated a discrete-time stochastic Hawk–Dove game. Our code is available on GitHub; we did not pursue analytical derivations. A Hawk competing against a Dove always obtains the contested resource and receives a benefit *B*; a Hawk competing with a Hawk either gains the benefit *B*, or experiences a cost of fighting *C*, with equal probability; competing Doves split the benefit *B* evenly. Each timestep, individuals die with probability *d* and then, if alive, produce offspring according to a Poisson distribution with mean $b_{0}\exp\;[s(\;p_{H}0.5(B-C)+p_{D}B)-\alpha N]$ for Hawks and $b_{0}\exp\;[s\;p_{D}0.5B-\alpha N]$ for Doves. Here, $b_{0}$ is the baseline birth rate, *s* is the intensity of selection, $p_{H}=1-p_{D}$ is the proportion of Hawks, *α* is a density dependence term, and *N* is the total abundance $N_{H}+N_{D}$. In all simulations, $d=0.01,\;b_{0}=0.05,\;\alpha =0.15,\;s=0.5,\;B=2,$ and 2.25≤C≤5.75. A stable polymorphism requires B<C. a) Hawks and Doves coexist (B=2,C=4.4) with abundances fluctuating around their mean field expectations (horizontal lines); we use a reflecting boundary to prevent chance extinction. The 3 components of our novel fitness operationalization are illustrated for the Hawk b) and Dove c), as they emerge from simulations. When a previously absent Hawk or Dove is introduced by mutation or migration, it must escape stochastic loss to establish. We operationalized establishment as reaching the mean field equilibrium abundance. Gray time series data depict failures to establish. After establishment (colored blue and green time series data), Hawk and Dove abundances fluctuate around their means ($N_{H}$ and $N_{D}$) for a sojourn time ($\tau_{H}$ and $\tau_{D}$) until eventual extinction. d) to f) each show a fitness component of Hawks relative to Doves, based on 7,500 simulations of a single Hawk introduced into a Dove population and vice versa. Each replicate simulation continues until the invader is lost; for simplicity, we apply a reflecting boundary to the resident. d) To calculate establishment and counter-establishment probabilities, we divided the number of establishments by 7,500. e) The ratio of mean sojourn times following establishment events. f) The ratio of mean abundances during the sojourns that followed establishment events. g) shows the time-integral (area under $N_{H}(t)$ and $N_{D}(t)$). For each replicate, we computed the the time-integral (here calculated over discretized time) of the invader's abundance from introduction until extinction. We then summed these integrals across all 7,500 replicates for each direction of invasion and took the ratio of the 2 pooled totals. This pooled time-integral ratio is negligibly different from the product of components d) to f).

Balancing selection is a challenge to all the derived operationalizations presented above. Sometimes 2 alleles can each invade an equilibrium population of the other, such that both variants are maintained by balancing selection (Fig. 3a). Identifying the conditions for *mutual invasibility* is common in evolutionary game theory (Maynard Smith and Price 1973) and adaptive dynamics (Metz et al. 1995). Mutual invasibility is also the central criterion for stable species coexistence in ecological models (Turelli 1978; Chesson 2000). When coexisting deterministically, both types have a geometric mean fitness of 1. With fixation of either being an atypical outcome, taking the ratio of fixation probabilities contains little information about the outcomes natural selection tends to produce.

The qualitative intuition that “both types are fit” can be operationalized in stochastic terms by noting that both types invade with a high probability of establishment. Focusing on establishment sidesteps the rarity of fixation. A “high” establishment probability can be operationalized by comparing an invader's probability of reaching a given, arbitrary frequency to that of a neutral reference invader (i.e. one indistinguishable from the resident).

To quantitatively operationalize fitness under balancing selection, we propose taking the time-integral of mutant lineage abundance from introduction into a resident population of the other type, until stochastic extinction. We then take the ratio of these integrals, switching which is the resident and which is the invader. This is illustrated in Fig. 3 for the Hawk–Dove game.

The time-integral is only slightly larger than the product of 3 informative components: establishment probability, sojourn time from introduction until extinction conditional on establishment, and mean abundance during its sojourn (Fig. 3b to g). Minor deviation of overall fitness from the product of these 3 components comes from neglecting abundance conditional on non-establishment. As a technical matter to prevent the sojourn time from being inflated by fixation events, a model should disallow transitions to the absorbing boundary of invader fixation. Our metric captures the potential vulnerability of an abundant type to extinction, e.g. from disturbance (Tilman et al. 1994), which would be missed if we used abundance or biomass (Van Valen 1976) in the corresponding mean field model.

Stable polymorphisms are important not just under the Hawk–Dove dynamics but also in temporally fluctuating environments. Four distinct mechanisms were reviewed by Bertram and Masel (2019b), each of which suggests different kinds of empirical model systems. Long-term observations are needed to explore the application of our time-integral metric, with lineage coexistence during experimental evolution (Good et al. 2017) potentially providing a particularly good study system.

## The role of fitness within evolution by natural selection

**Fig. 4.**
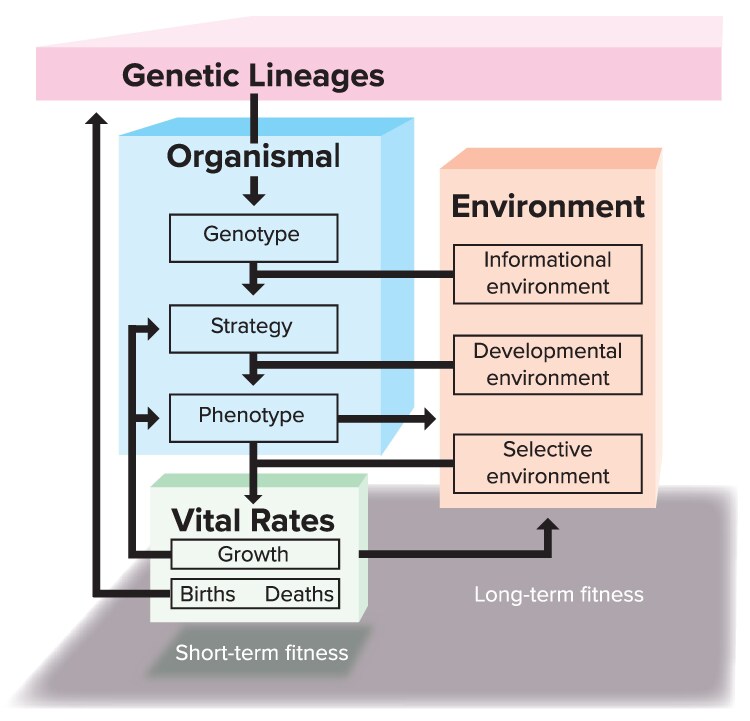
Causal diagram of the key components/factors underlying operationalizations of fitness. All arrows imply causality. Evolution by natural selection involves feedback between genes, environment, organismal phenotypes, and vital rates. Short-term fitness (e.g. relative per-generation fitness, absolute per-generation fitness, or the derived Malthusian parameter) summarizes current vital rates, whereas long-term, derived fitness operationalizations reflect the fate of genetic lineages within more complete feedback systems. Both are illustrated here as shadows, indicating projections in a mathematical sense. The environment experienced by an organism includes all abiotic factors (mean physical conditions, including the effects of biotic resource depletion and ecosystem engineering) and biotic factors (direct effects of conspecific and heterospecific abundances). All 3 vital rates (births, deaths, and organismal growth), as well as phenotypes, feed back to the environment, because population density and its consequences are important aspects of the environment. Genotypes and the informational environment (i.e. interpretable cues that organisms plastically respond to, via phenotypic plasticity and epigenetics) give rise to the strategies used by organisms. Strategies consist of investment allocations subject to life history trade-offs such as Grime's CSR triangle (Grime 1977), the competition–colonization trade-off (Tilman 1994), and bet hedging. Phenotypes emerge from strategies deployed within a developmental environment. Niche construction and migration phenotypes affect the environment, or which environment is experienced, respectively. Selection on phenotypes gives rise to differences in vital rates. While the authors differ in their metaphysical interpretations of this figure (i.e. whether the objects shown in 3D are in fact appropriately depicted as “real” objects with fitness as a mere shadow (Byerly and Michod 1991; Krimbas 2004), or whether the objects shown in 3D are rather themselves shadow-like, imperfect measures of fitness as a “real” property), what the figure shows regarding various considerations for operationalizing fitness and the relationships among alternative operationalizations is compatible with either metaphysical picture (Pence and Ramsey 2013; Walsh et al. 2017; Takacs and Bourrat 2021; Bourrat et al. 2024).

Figure 4 illustrates how models describe causality during evolution by natural selection. We distinguish between 3 aspects of the environment. The *selective environment* interacts with phenotypes to give rise to organisms' instantaneous vital rates. Note that our use of “selective environment” better corresponds to the “ecological environment” of Brandon (1990). Here, we restrict the term *phenotype* to realized organismal properties (e.g. body size) or behaviors (e.g. migration, aggression). Extended phenotypes (Dawkins 1982) are captured by feedback from phenotype to the environment (Fig. 4). We define aspects of the environment that exert direct causal influence on phenotypes as the *developmental environment*. Organismal *strategies* describe which phenotypes are pursued given constraints on what is possible (see section below for details). Strategies are shaped by genotype and/or by a plastic response to the *informational environment*—the cues that organisms respond to, prior to the direct effects of the environment on development. Any responses to the informational environment (e.g. using locally low resource levels as a cue to migrate) reflect the history of adaptation. In contrast, we consider intrinsic effects of the environment on phenotypes (e.g. reactions proceed faster at higher temperatures; Brown et al. 2004) to be part of the developmental environment. Note that the same environmental factor (e.g. temperature) can be part of all 3 aspects of the environment, by giving information, altering development, and imposing selection.

Different models simplify the Fig. 4 scheme in different ways. Commonly assigned fitness operationalizations, e.g. per-generation absolute fitness W=b/d, summarize the differential *vital rates* that embody natural selection in the short term (Fig. 4, small shadow). In the Wright–Fisher model, genotypes vary in *b*, whereas in Haldane's model and the Moran model (Moran 1958), they can also vary in *d*. Haldane holds the environment constant, whereas the Wright–Fisher model lets the selective environment (represented by allele frequencies) affect the absolute vital rate *b* of a given genotype.

More complex fitness operationalizations are then derived to summarize the longer-term fate of genetic lineages, including the influences of demographic stochasticity, migration, niche construction, and spatial and temporal environmental variation (Fig. 4, large shadow). Natural selection produces differential vital rates, whereas the long-term outcomes of natural selection are embodied in long-term lineage fate. Simple population genetic models that assign values of *s* can provide insights into the efficacy and timescale over which natural selection may operate, e.g. invasion probability $∼2s/\sigma^{2}$ and sojourn time ∼2(ln(sN)+γ)/s. However, operationalizations that directly assign fitness as an abstract parameter *s* (rather than deriving appropriate operationalization(s) of fitness from a genotype–phenotype–environment mapping) do not provide insights into the underlying biological mechanisms through which natural selection favors particular traits.

Directly assigning vital rates enables us to use models to ask, for example, how natural selection acts during the evolution of dormancy, when modeled as a genetically encoded 1-locus strategy to germinate with probability *g* per year. More sophisticated strategies might include active sensing to exploit the informational environment (Kussell and Leibler 2005). For example, selection might favor a reaction norm of higher *g* given higher soil moisture. A sufficiently reliable environmental cue begets a shift from bet hedging to plasticity (Botero et al. 2015). Selection acts on phenotypes (germinating vs not) as a function of both biotic environment (population density) and abiotic environment (drought vs non-drought year), to produce vital rates whose impact on genetic lineages, over time, can be summarized by derived fitness operationalizations. This type of model provides insights into the biological mechanism through which a lineage with a mutation ($g_{I}$) “wins.”

Fromhage et al. (2024) distinguish between 5 properties that have motivated fitness operationalizations: predictors of short-term (A) phenotypic change and (B) gene-frequency change, (C) “improvement” criteria, and performance measures of (D) phenotypic strategies and (E) individual organisms. Others, notably Autzen and Okasha (2022), emphasize (F) capturing the endpoint of the evolutionary process under natural selection. Here, we have emphasized assigning vital rates as performance measures of individual organisms (E) in order to derive lineage properties (D) that capture what natural selection favors (F). B is fulfilled by relative Malthusian fitness, a derived short-term fitness operationalization. The interpretation of Malthusian fitness or its extensions as invasion fitness is a short-term approximation of D. Fromhage et al. (2024) argue for the “folk definition of inclusive fitness” to address C. In contrast, we advocate for a diversity of design principles, rather than one universal design principle of “fitness.” Strategies play this role within our scheme. We do not claim that evolution by natural selection maximizes fitness in any of its operationalizations (Allen et al. 2013; Allen and Nowak 2016; Birch 2016)—we simply ask what strategies tend to evolve.

Social interactions are often treated as *the* key complication for defining fitness. For example, Fromhage et al.’s (2024) scheme is correspondingly focused on debates about the role of inclusive fitness, neglecting e.g. complications from non-overlapping generations. Inclusive fitness is a derived fitness operationalization, traditionally viewed as a short-term organismal property. However, the same inclusive fitness operationalization can be viewed as a lineage property, namely, the mean reproductive success of individuals across the probability distribution of lineage fates (Akçay and Van Cleve 2016). In our view, social interactions are simply one aspect of the density and frequency dependence of the biotic environment, and our same scheme of deriving lineage properties from organismal vital rates applies.

### Strategies

Strategies are intermediate between genotype and phenotype. In a broader sense, strategies are a form of phenotype, describing what an organism prioritizes given constraints, often entailing commitment to developmental pathways and/or behaviors. Strategies can be seen, albeit controversially, as setting organismal goals. The decision to commit is informed by genotype and by the informational environment, with its success in achieving the anticipated phenotype affected by the developmental environment.

As a simple example, consider a “Hawk” strategy from the Hawk–Dove game in evolutionary game theory (Maynard Smith and Price 1973). Hawks fight for resources, and Doves avoid conflict. In classic game theoretic models, the developmental environment is neglected, and having a Hawk strategy fully specifies behavioral phenotypes. One's opponent (Hawk or Dove) constitutes one's selective environment, and knowledge of their past behavior (if included in the model variant) constitutes the informational environment. In contrast, we conceptualize a Hawk *strategy* not just as behaviors within the narrow confines of game theory, but as a developmental commitment toward *developing a set of phenotypes* (both armaments and behaviors) that are relevant for implementing aggression. This allows for the possibility that developmental conditions (e.g. insufficient resources) may prevent a Hawk from e.g. achieving large enough body size or armaments to be successful. The individual may then switch strategies, treating developmental inputs as part of the informational environment.

Applying our distinction between strategy and phenotype to our seed bank example is more subtle. A seed's realized phenotype is defined by germination (or lack thereof) whereas its strategy is embodied in the stochastic gene circuitry that is an adaptation for achieving a probability of germination *g* within the historical range of environments. An organism's realized phenotype arises from the latter via noise within the developmental environment (Frank 2011b). An unanticipated developmental environment (e.g. a prolonged hard freeze) could cause the phenotypic outcome (germinating with probability *g*) to deviate from the strategy.

Strategies include investing in rapid growth given low population density, or in competitiveness or persistence given high population density (Grime 1988; Bertram and Masel 2019a). This was originally formalized as *r*- vs *K*-selected “strategies” (MacArthur 1962; Roughgarden 1971), where *r* is the Malthusian parameter at low density (and a prefactor of it also at higher densities), and *K* describes susceptibility to density dependence (similar to 1/α in equation (2)). A trade-off between investment in *r* vs *K* was assumed, with the resulting “strategy” reflecting an organism's position along that trade-off. However, *r* and *K* are often positively correlated with slope near 1 in empirical studies (Luckinbill 1978, 1979; Valle et al. 1989; Kuno 1991; Hendriks et al. 2005; Fitzsimmons et al. 2010), in agreement with some process-based theoretical models (Travis et al. 2023). While there does seem to be a fast–slow continuum, contemporary life history theory also categorizes strategies in other ways (Salguero-Gómez et al. 2016; Healy et al. 2019; Malik et al. 2020; Bruggeman et al. 2023; Stott et al. 2024).

Organisms are capable of an extraordinary variety of phenotypes. The “functional trait” literature in community ecology attempts to reduce this dimensionality by focusing on phenotypes (e.g. wood density, seed size, metabolic rate) that are most closely tied to strategies and vital rates (McGill et al. 2006; Yang et al. 2018). In contrast, vital rates come in only 3 key varieties, applied to different life history stages. Organismal strategies might have far lower dimensionality than downstream functional traits or other organismal phenotypes, in a manner that helps provide generalizable insights. Strategy space might be both small enough and concrete enough to make the organism's developmental commitments coherent and thus make the study of phenotypes tractable. Assigned and derived fitness operationalizations are key components of the models that serve to clarify how natural selection acts on strategies.

We find the 3-dimensional scheme of Grime to be a promising starting point for characterizing strategies with respect to population density. Grime (1988, 1977, 2001) hypothesized that trade-offs shape species into 3 types of specialization—“ruderals” tolerate harsh abiotic environments, “competitors” excel at high population density, and “colonizers” rapidly disperse to ephemeral resources. Each strategy is closely tied to vital rates. High-dimensional phenotype space among e.g. coral species (Darling et al. 2012) and plant species (Liu et al. 2025) can be simplified via a space of just these 3 strategies. Our simple example of a seed bank illustrates how organismal strategies can be described with reference to vital rates (and potentially also migration and niche construction phenotypes) in order to gain insight into how populations evolve within strategy space.

## Conclusion

Both genotype space and phenotype space are huge and must be simplified to produce generalizable biological insight. The simplest, most traditional population genetic models, which appear to assign relative or absolute fitness values to genotypes, in fact implicitly assign organismal vital rates. More complex models are often used to investigate which organismal “strategies” (e.g. aggression or dormancy) are expected to evolve under which circumstances. Strategies may depend on environmental information as well as on genotype. Models of biological strategies assign vital rates and sometimes also migration rates and niche construction phenotypes. From these assignations, a greater variety of metrics are derived to quantify what natural selection favors than is commonly appreciated. Which metric is appropriate depends on the exact biological question. For example, the Malthusian parameter and its variations capture adaptation speed, which is important for response to disturbances. When rare mutations and/or fluctuating environments cause lineages to be lost stochastically, a different approach may be needed, e.g. using the fixation: counterfixation probability ratio. We build on the latter to propose a new, lineage-based fitness operationalization suitable for describing fitness under balancing selection.

## Data Availability

No data were produced in this study. Code is available at https://github.com/DanielSmithEcology/Fitness_Definitions_Code.
